# Tris(tetra­butyl­ammonium) hexa­kis­(*tert*-butane­thiol­ato-κ*S*)hepta-μ_3_-chlorido-μ_3_-sulfido-hexa­molybdate dihydrate

**DOI:** 10.1107/S1600536812007416

**Published:** 2012-02-24

**Authors:** Pavel A. Petrov, Dmitry Yu. Naumov, Sergey N. Konchenko

**Affiliations:** a Nikolaev Institute of Inorganic Chemistry, SB Russian Academy of Sciences, Akademician Lavrentiev prospekt 3, Novosibirsk 90, 630090, Russian Federation, and, Novosibirsk State University, Pirogov street 2, Novosibirsk 90, 630090, Russian Federation

## Abstract

The octa­hedral cluster core of the anion in the structure of the title compound, (C_16_H_36_N)_3_[Mo_6_(C_4_H_9_S)_6_(μ_3_-Cl)_7_(μ_3_-S)]·2H_2_O, has -3 site symmetry. Two μ_3_-Cl atoms fully occupy positions in the cluster core, while the remaining six positions are statistically occupied by Cl and S atoms in a 1:5 ratio. The fully occupied Cl-atom positions are located on sites with 3 symmetry, and the N atom of tetra­butyl­ammonium cation is located on a site with 2 symmetry. The structure contains also two disordered solvent water mol­ecules, one of which is located on a threefold rotation axis and the other in a general position, both with an occupancy of 0.25. The water mol­ecules are localized in cavities formed by the tetra­butyl­ammonium cations and the *tert*-butane­thiol­ate groups. The metal clusters are stacked in a cubic close packing arrangement along [001].

## Related literature

For a review of octa­hedral halogen-bridged metal clusters, see: Prokopuk & Shryver (1998 ▶). For synthesis and structures of related halogen/chalcogen clusters, see: Abramov *et al.* (2009 ▶); Ebihara *et al.* (1988 ▶); Ebihara, Imai *et al.* (1995 ▶); Ebihara, Toriumi *et al.* (1995 ▶); Michel & McCarley (1982 ▶); Nocera & Gray (1984 ▶). For a related transformation of *t*BuS^−^, see: Petrov *et al.* (2010 ▶). For synthesis and structures of related clusters with sulfur-substituted halogen atoms, see: Schoonover *et al.* (1996 ▶); Szczepura *et al.* (2008 ▶).
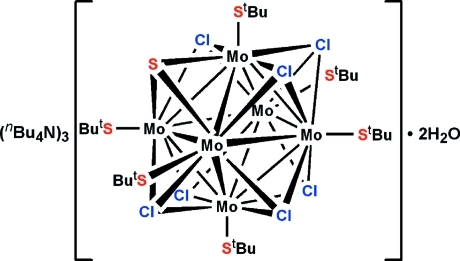


## Experimental

### 

#### Crystal data

(C_16_H_36_N)_3_[Mo_6_(C_4_H_9_S)_6_Cl_7_S]·2H_2_O
*M* *_r_* = 2154.29
Trigonal, 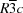

*a* = 18.7481 (5) Å
*c* = 52.4233 (12) Å
*V* = 15957.7 (7) Å^3^
*Z* = 6
Mo *K*α radiation
μ = 1.04 mm^−1^
*T* = 150 K
0.42 × 0.35 × 0.23 mm

#### Data collection

Bruker–Nonius X8 APEX CCD diffractometer
Absorption correction: multi-scan (*SADABS*; Bruker, 2004 ▶) *T*_min_ = 0.670, *T*_max_ = 0.797
36925 measured reflections
3637 independent reflections
3092 reflections with *I* > 2σ(*I*)
*R*_int_ = 0.034

#### Refinement

*R*[*F* ^2^ > 2σ(*F* ^2^)] = 0.038
*wR*(*F* ^2^) = 0.125
*S* = 1.14
3637 reflections
161 parameters
12 restraints
H-atom parameters constrained
Δρ_max_ = 1.16 e Å^−3^
Δρ_min_ = −0.76 e Å^−3^

### 

Data collection: *APEX2* (Bruker, 2004 ▶); cell refinement: *SAINT* (Bruker, 2004 ▶); data reduction: *SAINT*; program(s) used to solve structure: *SHELXS97* (Sheldrick, 2008 ▶); program(s) used to refine structure: *SHELXL97* (Sheldrick, 2008 ▶); molecular graphics: *SHELXTL* (Sheldrick, 2008 ▶), *Mercury* (Macrae *et al.*, 2006 ▶) and *POV-RAY* (Persistence of Vision, 2004 ▶); software used to prepare material for publication: *SHELXTL*.

## Supplementary Material

Crystal structure: contains datablock(s) I, global. DOI: 10.1107/S1600536812007416/wm2589sup1.cif

Structure factors: contains datablock(s) I. DOI: 10.1107/S1600536812007416/wm2589Isup2.hkl

Additional supplementary materials:  crystallographic information; 3D view; checkCIF report

## Figures and Tables

**Table 1:** Selected bond lengths (Å)

Mo1—Mo1^i^	2.6067 (4)
Mo1—Mo1^ii^	2.6328 (5)
Mo1—S1	2.5158 (9)
Mo1—S2^iii^	2.4792 (9)
Mo1—S2^iv^	2.4842 (9)
Mo1—Cl1	2.5054 (10)
Mo1—Cl2	2.4801 (9)
Mo1—Cl2^iii^	2.4792 (9)
Mo1—Cl2^iv^	2.4842 (9)

Symmetry codes: (i) 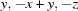; (ii) 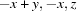; (iii) 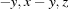; (iv) 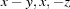.

**Fig. 1.**
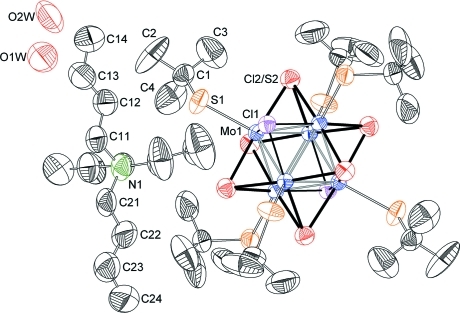
Molecular structure of [Mo6(µ3-S)(µ3-Cl)7(StBu)6]3- anion. Displacement ellipsoids are plotted at the 50% probability level.

**Fig. 2.**
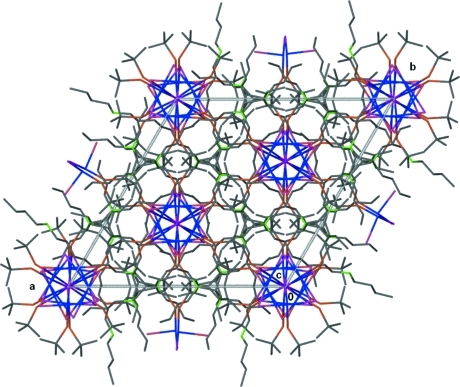
Packing of the structure viewed along c axis. O and H atoms are omitted for clarity.

## supplementary crystallographic information

### Comment

The octahedral clusters of early transition metals are often regarded as precursors of functional materials with redox and/or luminescent properties. The advantage of halide-bridged clusters [Mo_6_(µ_3_-*X*)_8_*X*_6_]^2-^ (*X* = halogen) is the ability of tuning the electronic structure and the properties of the cluster core by step-by-step exchange of the terminal *X* atoms. The exchange of the µ_3_-bridging *X* atoms is also possible; however, reactions of this type are less common. The halogen-chalcogen clusters with one or two chalcogen atoms (or their mixtures) were obtained in the reactions of [Mo_6_(µ_3_-*X*)_8_*X*_6_]^2-^ with NaSH, NaSeH or *in situ*-generated H_2_Se (Michel & McCarley, 1982; Ebihara *et al.*, 1988; Ebihara, Imai *et al.*, 1995; Ebihara, Toriumi *et al.*, 1995; Abramov *et al.*, 2009).

Recently, the reaction of [Mo_6_(µ_3_-*X*)_8_(OMe)_6_]^2-^ with excess EtSH was reported leading to the smooth substitution of the terminal methoxides to ethanethiolate groups. The latter can be further substituted by other S*R*^-^ groups where *R* = butyl, benzyl or 3-indolyl (Szczepura *et al.*, 2008). Our attempt was aimed to prove if the reaction of [Mo_6_(µ_3_-*X*)_8_*X*_6_]^2-^ with *^t^*BuSNa would stop on the substitution of the terminal *X* atoms, or would result in a core rearrangement as well. Previously, *^t^*BuS^-^ was reported to be the source of the S^2-^ anion (see, for example: Petrov *et al.* 2010).

The presence of three tetrabutylammonium cations designates the charge of the cluster core. Keeping in mind the high oxidation potential of the [Mo_6_(µ_3_-Cl)_8_Cl_6_]^3-/2-^ pair (1.53 V in MeCN *versus* SCE, Nocera & Gray, 1984) one would formulate the cluster core composition as [Mo_6_(µ_3_-S)(µ_3_-Cl)_7_(S*^t^*Bu)_6_]^3-^. The analysis of the temperature factors of the atoms in the µ_3_-positions leads us to the conclusion that two positions are occupied with Cl atoms only, while the remaining six positions are statistically occupied with Cl and S atoms in a 1:5 ratio (Fig. 1). The presence of one S atom in the cluster core has no noticeable effect on its geometry (Schoonover *et al.*, 1996; Szczepura *et al.*, 2008).

The structure contains two disordered lattice water molecules. One is located on a threefold rotation axis, the other is located in a general position. Both have an occupancy of 0.25 and are disordered over a site with symmetry 32. These two water molecules have an O ··· O distance of 2.706 (8) Å, pointing to hydrogen-bonding interactions. The water molecules are localized in cavities formed by the Bu_4_N^+^ cations and *^t^*BuS groups. The water incorporated in the structure most likely originated from the starting material (Bu_4_N)_2_[Mo_6_(µ_3_-Cl)_8_Cl_6_]^.^*n*H_2_O.

The centres of the metal clusters are arranged in a cubic close packing along [001] as stacking direction (Fig. 2).

### Experimental

A mixture of 128.8 mg (0.083 mmol) (Bu_4_N)_2_[Mo_6_Cl_14_] and 154.8 mg (1.38 mmol) NaS*^t^*Bu (1:16.7 molar ratio) in 15 ml CH_3_CN was refluxed for 5 days. The resulting brown solution was filtered to remove the white residue and left standing at 278 K. After several weeks almost black crystals were formed. The largest positive and negative residual electron densities are located 0.73 Å from atom OW3 and 0.58 Å from atom S2, respectively.

### Refinement

The site occupation factors of the S and Cl atoms of the disordered Cl2/S2 site were preliminary refined without any constrains giving us the ratio. Constrained occupation factors were taken into account in the final refinement cycle. The composition of the anion has been confirmed by electrospray mass-spectrometry. The signal at m/*z* 695.7 was assigned to the [Mo_6_(µ_3_-S)(µ_3_-Cl)_7_(S*^t^*Bu)_6_]]^2-^ anion. The H atoms of the disordered water molecules could not be located and were excluded from refinement.

### Crystal data

**Table:**

(C_16_H_36_N)_3_[Mo_6_(C_4_H_9_S)_6_Cl_7_S]·2H_2_O	*D*_x_ = 1.345 Mg m^−^^3^
*M**_r_* = 2154.29	Mo *K*α radiation, λ = 0.71073 Å
Trigonal, *R*3*c*	Cell parameters from 9844 reflections
Hall symbol: -R 3 2"c	θ = 2.5–28.3°
*a* = 18.7481 (5) Å	µ = 1.04 mm^−^^1^
*c* = 52.4233 (12) Å	*T* = 150 K
*V* = 15957.7 (7) Å^3^	Prism, brown
*Z* = 6	0.42 × 0.35 × 0.23 mm
*F*(000) = 6708	

### Data collection

**Table:**

Bruker–Nonius X8 APEX CCD diffractometer	3637 independent reflections
Radiation source: fine-focus sealed tube	3092 reflections with *I* > 2σ(*I*)
Graphite monochromator	*R*_int_ = 0.034
Detector resolution: 25 pixels mm^-1^	θ_max_ = 26.4°, θ_min_ = 2.2°
φ scans	*h* = −23→23
Absorption correction: multi-scan (*SADABS*; Bruker, 2004)	*k* = −23→22
*T*_min_ = 0.670, *T*_max_ = 0.797	*l* = −65→55
36925 measured reflections	

### Refinement

**Table:**

Refinement on *F*^2^	Primary atom site location: structure-invariant direct methods
Least-squares matrix: full	Secondary atom site location: difference Fourier map
*R*[*F*^2^ > 2σ(*F*^2^)] = 0.038	Hydrogen site location: inferred from neighbouring sites
*wR*(*F*^2^) = 0.125	H-atom parameters constrained
*S* = 1.14	*w* = 1/[σ^2^(*F*_o_^2^) + (0.0654*P*)^2^ + 69.7786*P*] where *P* = (*F*_o_^2^ + 2*F*_c_^2^)/3
3637 reflections	(Δ/σ)_max_ < 0.001
161 parameters	Δρ_max_ = 1.16 e Å^−^^3^
12 restraints	Δρ_min_ = −0.76 e Å^−^^3^

### Special details

**Table:**

Geometry. All e.s.d.'s (except the e.s.d. in the dihedral angle between two l.s. planes) are estimated using the full covariance matrix. The cell e.s.d.'s are taken into account individually in the estimation of e.s.d.'s in distances, angles and torsion angles; correlations between e.s.d.'s in cell parameters are only used when they are defined by crystal symmetry. An approximate (isotropic) treatment of cell e.s.d.'s is used for estimating e.s.d.'s involving l.s. planes.
Refinement. Refinement of *F*^2^ against ALL reflections. The weighted *R*-factor *wR* and goodness of fit *S* are based on *F*^2^, conventional *R*-factors *R* are based on *F*, with *F* set to zero for negative *F*^2^. The threshold expression of *F*^2^ > σ(*F*^2^) is used only for calculating *R*-factors(gt) *etc*. and is not relevant to the choice of reflections for refinement. *R*-factors based on *F*^2^ are statistically about twice as large as those based on *F*, and *R*- factors based on ALL data will be even larger.Hydrogen atoms of water molecules are not located. One of water molecules is disodered by two positions. Hydrogen atoms of cation and anion are placed geometrically.

### Fractional atomic coordinates and isotropic or equivalent isotropic displacement parameters (Å^2^)

**Table:**

	*x*	*y*	*z*	*U*_iso_*/*U*_eq_	Occ. (<1)
Mo1	0.093280 (16)	0.039723 (17)	0.020197 (5)	0.02512 (13)	
Cl1	0.0000	0.0000	0.05819 (2)	0.0298 (3)	
Cl2	0.07472 (5)	−0.10085 (5)	0.019692 (16)	0.0374 (2)	0.8333333
S1	0.21632 (6)	0.09894 (7)	0.049357 (18)	0.0458 (3)	
S2	0.07472 (5)	−0.10085 (5)	0.019692 (16)	0.0374 (2)	0.1666667
O2W	0.5911 (10)	0.3102 (13)	0.0925 (4)	0.103 (6)	0.25
N1	0.2617 (3)	0.3333	0.0833	0.0555 (12)	
C1	0.3076 (2)	0.0927 (3)	0.03926 (8)	0.0478 (9)	
C2	0.3649 (4)	0.1194 (6)	0.06188 (11)	0.107 (3)	
H2A	0.4128	0.1137	0.0578	0.161*	
H2B	0.3359	0.0848	0.0766	0.161*	
H2C	0.3831	0.1771	0.0659	0.161*	
C3	0.2844 (4)	0.0089 (4)	0.02944 (17)	0.111 (3)	
H3A	0.2454	−0.0054	0.0153	0.167*	
H3B	0.2587	−0.0316	0.0432	0.167*	
H3C	0.3339	0.0089	0.0234	0.167*	
C4	0.3539 (4)	0.1528 (5)	0.01793 (13)	0.099 (2)	
H4A	0.3731	0.2091	0.0238	0.148*	
H4B	0.3172	0.1408	0.0033	0.148*	
H4C	0.4013	0.1473	0.0129	0.148*	
C11	0.3094 (3)	0.3354 (3)	0.10717 (8)	0.0559 (11)	
H11A	0.3620	0.3883	0.1075	0.067*	
H11B	0.2773	0.3342	0.1223	0.067*	
C12	0.3281 (3)	0.2659 (3)	0.10940 (9)	0.0669 (13)	
H12A	0.2761	0.2126	0.1078	0.080*	
H12B	0.3645	0.2697	0.0952	0.080*	
C13	0.3688 (4)	0.2676 (3)	0.13419 (10)	0.0809 (16)	
H13A	0.3314	0.2620	0.1484	0.097*	
H13B	0.4195	0.3218	0.1360	0.097*	
C14	0.3909 (4)	0.2012 (4)	0.13669 (13)	0.0886 (18)	
H14A	0.3405	0.1473	0.1369	0.133*	
H14B	0.4214	0.2090	0.1526	0.133*	
H14C	0.4252	0.2040	0.1222	0.133*	
C21	0.2550 (3)	0.4108 (3)	0.08358 (10)	0.0649 (13)	
H21A	0.3108	0.4587	0.0862	0.078*	
H21B	0.2212	0.4081	0.0984	0.078*	
C22	0.2179 (5)	0.4266 (4)	0.05962 (15)	0.107 (3)	
H22A	0.2472	0.4229	0.0444	0.128*	
H22B	0.1595	0.3828	0.0582	0.128*	
C23	0.2222 (4)	0.5059 (4)	0.05953 (12)	0.0854 (17)	
H23A	0.2808	0.5494	0.0606	0.102*	
H23B	0.1942	0.5099	0.0750	0.102*	
C24	0.1854 (6)	0.5224 (5)	0.03716 (18)	0.136 (4)	
H24A	0.2026	0.5059	0.0216	0.204*	
H24B	0.2039	0.5814	0.0364	0.204*	
H24C	0.1252	0.4910	0.0386	0.204*	
O1W	0.6667	0.3333	0.0468 (7)	0.095 (8)	0.25

### Atomic displacement parameters (Å^2^)

**Table:**

	*U*^11^	*U*^22^	*U*^33^	*U*^12^	*U*^13^	*U*^23^
Mo1	0.02590 (17)	0.02668 (17)	0.02253 (19)	0.01297 (12)	−0.00049 (9)	−0.00092 (9)
Cl1	0.0327 (4)	0.0327 (4)	0.0241 (6)	0.0163 (2)	0.000	0.000
Cl2	0.0400 (4)	0.0387 (4)	0.0356 (4)	0.0212 (4)	−0.0011 (3)	0.0002 (3)
S1	0.0345 (5)	0.0652 (6)	0.0409 (5)	0.0273 (4)	−0.0101 (4)	−0.0199 (4)
S2	0.0400 (4)	0.0387 (4)	0.0356 (4)	0.0212 (4)	−0.0011 (3)	0.0002 (3)
O2W	0.049 (7)	0.116 (10)	0.122 (10)	0.024 (6)	0.007 (6)	0.007 (7)
N1	0.060 (2)	0.051 (3)	0.052 (3)	0.0254 (14)	−0.0121 (11)	−0.024 (2)
C1	0.0364 (19)	0.054 (2)	0.057 (2)	0.0256 (18)	−0.0009 (17)	−0.0042 (18)
C2	0.061 (3)	0.210 (8)	0.076 (4)	0.086 (5)	−0.021 (3)	−0.020 (4)
C3	0.061 (3)	0.066 (4)	0.219 (8)	0.040 (3)	0.019 (4)	−0.011 (4)
C4	0.055 (3)	0.126 (6)	0.100 (5)	0.034 (4)	0.019 (3)	0.031 (4)
C11	0.062 (3)	0.056 (3)	0.044 (2)	0.024 (2)	−0.0055 (19)	−0.0146 (18)
C12	0.082 (3)	0.063 (3)	0.058 (3)	0.039 (3)	−0.011 (2)	−0.015 (2)
C13	0.110 (5)	0.063 (3)	0.063 (3)	0.038 (3)	−0.013 (3)	−0.002 (2)
C14	0.100 (5)	0.088 (4)	0.082 (4)	0.050 (4)	−0.012 (3)	0.009 (3)
C21	0.061 (3)	0.057 (3)	0.075 (3)	0.028 (2)	−0.019 (2)	−0.033 (2)
C22	0.131 (6)	0.086 (4)	0.122 (5)	0.070 (4)	−0.078 (5)	−0.058 (4)
C23	0.086 (4)	0.081 (4)	0.094 (4)	0.045 (3)	−0.018 (3)	−0.026 (3)
C24	0.157 (8)	0.091 (5)	0.181 (8)	0.078 (5)	−0.083 (7)	−0.039 (5)
O1W	0.086 (8)	0.086 (8)	0.113 (12)	0.043 (4)	0.000	0.000

### Geometric parameters (Å, º)

**Table:**

Mo1—Mo1^i^	2.6067 (4)	C3—H3C	0.9800
Mo1—Mo1^ii^	2.6067 (4)	C4—H4A	0.9800
Mo1—Mo1^iii^	2.6328 (5)	C4—H4B	0.9800
Mo1—Mo1^iv^	2.6328 (5)	C4—H4C	0.9800
Mo1—S1	2.5158 (9)	C11—C12	1.515 (7)
Mo1—S2^iv^	2.4792 (9)	C11—H11A	0.9900
Mo1—S2^ii^	2.4842 (9)	C11—H11B	0.9900
Mo1—Cl1	2.5054 (10)	C12—C13	1.500 (7)
Mo1—Cl2	2.4801 (9)	C12—H12A	0.9900
Mo1—Cl2^iv^	2.4792 (9)	C12—H12B	0.9900
Mo1—Cl2^ii^	2.4842 (9)	C13—C14	1.501 (8)
Cl1—Mo1^iii^	2.5054 (10)	C13—H13A	0.9900
Cl1—Mo1^iv^	2.5054 (10)	C13—H13B	0.9900
Cl2—Mo1^iii^	2.4792 (9)	C14—H14A	0.9800
Cl2—Mo1^i^	2.4842 (9)	C14—H14B	0.9800
S1—C1	1.849 (4)	C14—H14C	0.9800
O2W—O2W^v^	1.22 (4)	C21—C22	1.535 (8)
N1—C21^v^	1.520 (5)	C21—H21A	0.9900
N1—C21	1.520 (5)	C21—H21B	0.9900
N1—C11^v^	1.525 (5)	C22—C23	1.448 (8)
N1—C11	1.525 (5)	C22—H22A	0.9900
C1—C3	1.497 (7)	C22—H22B	0.9900
C1—C2	1.508 (6)	C23—C24	1.470 (9)
C1—C4	1.515 (7)	C23—H23A	0.9900
C2—H2A	0.9800	C23—H23B	0.9900
C2—H2B	0.9800	C24—H24A	0.9800
C2—H2C	0.9800	C24—H24B	0.9800
C3—H3A	0.9800	C24—H24C	0.9800
C3—H3B	0.9800		
			
S2^iv^—Mo1—Cl2^iv^	0.00 (5)	C2—C1—C4	106.6 (5)
S2^iv^—Mo1—Cl2	175.69 (3)	C3—C1—S1	111.9 (3)
Cl2^iv^—Mo1—Cl2	175.69 (3)	C2—C1—S1	106.4 (3)
S2^iv^—Mo1—S2^ii^	90.61 (2)	C4—C1—S1	111.6 (4)
Cl2^iv^—Mo1—S2^ii^	90.61 (2)	C1—C2—H2A	109.5
Cl2—Mo1—S2^ii^	90.59 (2)	C1—C2—H2B	109.5
S2^iv^—Mo1—Cl2^ii^	90.61 (2)	H2A—C2—H2B	109.5
Cl2^iv^—Mo1—Cl2^ii^	90.61 (2)	C1—C2—H2C	109.5
Cl2—Mo1—Cl2^ii^	90.59 (2)	H2A—C2—H2C	109.5
S2^ii^—Mo1—Cl2^ii^	0.00 (5)	H2B—C2—H2C	109.5
S2^iv^—Mo1—Cl1	89.24 (2)	C1—C3—H3A	109.5
Cl2^iv^—Mo1—Cl1	89.24 (2)	C1—C3—H3B	109.5
Cl2—Mo1—Cl1	89.22 (2)	H3A—C3—H3B	109.5
S2^ii^—Mo1—Cl1	175.32 (3)	C1—C3—H3C	109.5
Cl2^ii^—Mo1—Cl1	175.32 (3)	H3A—C3—H3C	109.5
S2^iv^—Mo1—S1	89.10 (3)	H3B—C3—H3C	109.5
Cl2^iv^—Mo1—S1	89.10 (3)	C1—C4—H4A	109.5
Cl2—Mo1—S1	94.93 (3)	C1—C4—H4B	109.5
S2^ii^—Mo1—S1	94.79 (3)	H4A—C4—H4B	109.5
Cl2^ii^—Mo1—S1	94.79 (3)	C1—C4—H4C	109.5
Cl1—Mo1—S1	89.88 (3)	H4A—C4—H4C	109.5
S2^iv^—Mo1—Mo1^i^	119.05 (2)	H4B—C4—H4C	109.5
Cl2^iv^—Mo1—Mo1^i^	119.05 (2)	C12—C11—N1	115.2 (3)
Cl2—Mo1—Mo1^i^	58.40 (2)	C12—C11—H11A	108.5
S2^ii^—Mo1—Mo1^i^	58.22 (2)	N1—C11—H11A	108.5
Cl2^ii^—Mo1—Mo1^i^	58.22 (2)	C12—C11—H11B	108.5
Cl1—Mo1—Mo1^i^	117.960 (18)	N1—C11—H11B	108.5
S1—Mo1—Mo1^i^	138.62 (2)	H11A—C11—H11B	107.5
S2^iv^—Mo1—Mo1^ii^	58.41 (2)	C13—C12—C11	112.5 (4)
Cl2^iv^—Mo1—Mo1^ii^	58.41 (2)	C13—C12—H12A	109.1
Cl2—Mo1—Mo1^ii^	119.04 (2)	C11—C12—H12A	109.1
S2^ii^—Mo1—Mo1^ii^	58.25 (2)	C13—C12—H12B	109.1
Cl2^ii^—Mo1—Mo1^ii^	58.25 (2)	C11—C12—H12B	109.1
Cl1—Mo1—Mo1^ii^	117.960 (17)	H12A—C12—H12B	107.8
S1—Mo1—Mo1^ii^	134.36 (3)	C12—C13—C14	114.0 (5)
Mo1^i^—Mo1—Mo1^ii^	60.665 (13)	C12—C13—H13A	108.8
S2^iv^—Mo1—Mo1^iii^	117.95 (2)	C14—C13—H13A	108.8
Cl2^iv^—Mo1—Mo1^iii^	117.95 (2)	C12—C13—H13B	108.8
Cl2—Mo1—Mo1^iii^	57.92 (2)	C14—C13—H13B	108.8
S2^ii^—Mo1—Mo1^iii^	117.86 (2)	H13A—C13—H13B	107.7
Cl2^ii^—Mo1—Mo1^iii^	117.86 (2)	C13—C14—H14A	109.5
Cl1—Mo1—Mo1^iii^	58.302 (15)	C13—C14—H14B	109.5
S1—Mo1—Mo1^iii^	135.38 (3)	H14A—C14—H14B	109.5
Mo1^i^—Mo1—Mo1^iii^	59.667 (7)	C13—C14—H14C	109.5
Mo1^ii^—Mo1—Mo1^iii^	90.0	H14A—C14—H14C	109.5
S2^iv^—Mo1—Mo1^iv^	57.95 (2)	H14B—C14—H14C	109.5
Cl2^iv^—Mo1—Mo1^iv^	57.95 (2)	N1—C21—C22	116.1 (4)
Cl2—Mo1—Mo1^iv^	117.91 (2)	N1—C21—H21A	108.3
S2^ii^—Mo1—Mo1^iv^	117.89 (2)	C22—C21—H21A	108.3
Cl2^ii^—Mo1—Mo1^iv^	117.89 (2)	N1—C21—H21B	108.3
Cl1—Mo1—Mo1^iv^	58.302 (15)	C22—C21—H21B	108.3
S1—Mo1—Mo1^iv^	131.38 (2)	H21A—C21—H21B	107.4
Mo1^i^—Mo1—Mo1^iv^	90.0	C23—C22—C21	113.7 (5)
Mo1^ii^—Mo1—Mo1^iv^	59.667 (7)	C23—C22—H22A	108.8
Mo1^iii^—Mo1—Mo1^iv^	60.0	C21—C22—H22A	108.8
Mo1^iii^—Cl1—Mo1^iv^	63.40 (3)	C23—C22—H22B	108.8
Mo1^iii^—Cl1—Mo1	63.40 (3)	C21—C22—H22B	108.8
Mo1^iv^—Cl1—Mo1	63.40 (3)	H22A—C22—H22B	107.7
Mo1^iii^—Cl2—Mo1	64.13 (2)	C22—C23—C24	115.3 (5)
Mo1^iii^—Cl2—Mo1^i^	63.36 (2)	C22—C23—H23A	108.4
Mo1—Cl2—Mo1^i^	63.35 (2)	C24—C23—H23A	108.4
C1—S1—Mo1	118.11 (13)	C22—C23—H23B	108.4
C21^v^—N1—C21	111.8 (5)	C24—C23—H23B	108.4
C21^v^—N1—C11^v^	107.2 (2)	H23A—C23—H23B	107.5
C21—N1—C11^v^	110.3 (3)	C23—C24—H24A	109.5
C21^v^—N1—C11	110.3 (3)	C23—C24—H24B	109.5
C21—N1—C11	107.2 (2)	H24A—C24—H24B	109.5
C11^v^—N1—C11	110.1 (5)	C23—C24—H24C	109.5
C3—C1—C2	113.8 (5)	H24A—C24—H24C	109.5
C3—C1—C4	106.5 (5)	H24B—C24—H24C	109.5

Symmetry codes: (i) *y*, −*x*+*y*, −*z*; (ii) *x*−*y*, *x*, −*z*; (iii) −*x*+*y*, −*x*, *z*; (iv) −*y*, *x*−*y*, *z*; (v) *x*−*y*+1/3, −*y*+2/3, −*z*+1/6.
